# Hyperuricaemia is associated with smaller volumes in the caudate nucleus head and putamen

**DOI:** 10.1093/braincomms/fcaf263

**Published:** 2025-07-15

**Authors:** Naoki Omori, Manabu Ishida, Masahiro Takamura, Satoshi Abe, Atsushi Nagai

**Affiliations:** Department of Neurology, Shimane University, Izumo City, Shimane 693-8501, Japan; Department of Research and Development, ERISA Corporation, Matsue City, Shimane 690-0816, Japan; Institutional Research Center, Fujita Health University, Toyoake City, Aichi 470-1192, Japan; Department of Neurology, Shimane University, Izumo City, Shimane 693-8501, Japan; Department of Neurology, Shimane University, Izumo City, Shimane 693-8501, Japan

**Keywords:** uric acid, hyperuricaemia, Basal ganglia, Parkinson’s disease, oxidative stress

## Abstract

Hyperuricaemia is a risk factor for gout, kidney diseases, and cerebrovascular diseases. Uric acid (UA) is also known as an antioxidant and has been suggested to inhibit the progression of neurodegenerative diseases, such as Parkinson’s disease. However, only a few studies have focused on the potential effects of UA on the basal ganglia. This study aimed to measure UA levels and basal ganglia volumes in community-dwelling adults and to evaluate the association thereof. Blood UA levels and brain MRI data were collected from individuals who underwent brain health checkups between January 2015 and March 2022 at Shimane Institute of Health Science. Participants were classified into three groups based on their UA levels: normal-low UA (< 5.0 mg/dL), normal-high UA (5.0–7.0 mg/dL), and hyperuricaemia (> 7.0 mg/dL). MRI was used to assess the presence of asymptomatic infarcts, microbleeds, and the severity of enlarged perivascular spaces in the basal ganglia. The volumes of the caudate nucleus, globus pallidus, putamen, substantia nigra, and subthalamic nucleus were calculated using voxel-based morphometry (VBM) as *Z*-scores adjusted for participant age, sex, and total intracranial volume. Analysis of covariance and smooth-curve fitting models were used to examine the association between UA levels and basal ganglia volumes. In total, 981 participants were included in the analysis. Analysis of covariance revealed that the hyperuricaemia group had significantly higher *Z*-scores (indicating smaller volumes) in the bilateral caudate nucleus head and putamen than those of the normal-high UA group. The smooth-curve model showed U-shaped associations with smaller volumes for both high and low UA levels, whereas piecewise linear regression analysis confirmed significant regression lines only in the group with higher UA levels. Although UA is thought to have a neuroprotective effect in adults, our findings indicate that hyperuricaemia may contribute to smaller volumes in the caudate nucleus head and putamen. This suggests that excessive UA levels could negatively affect basal ganglia structure and neurological health.

## Teaser

Omori *et al*. report that hyperuricaemia is associated with smaller volumes in the caudate nucleus head and putamen. Although uric acid is considered neuroprotective, these findings suggest that elevated levels may rather exert a negative influence on basal ganglia structure.

## Graphical Abstract

**Graphical Abstract:**
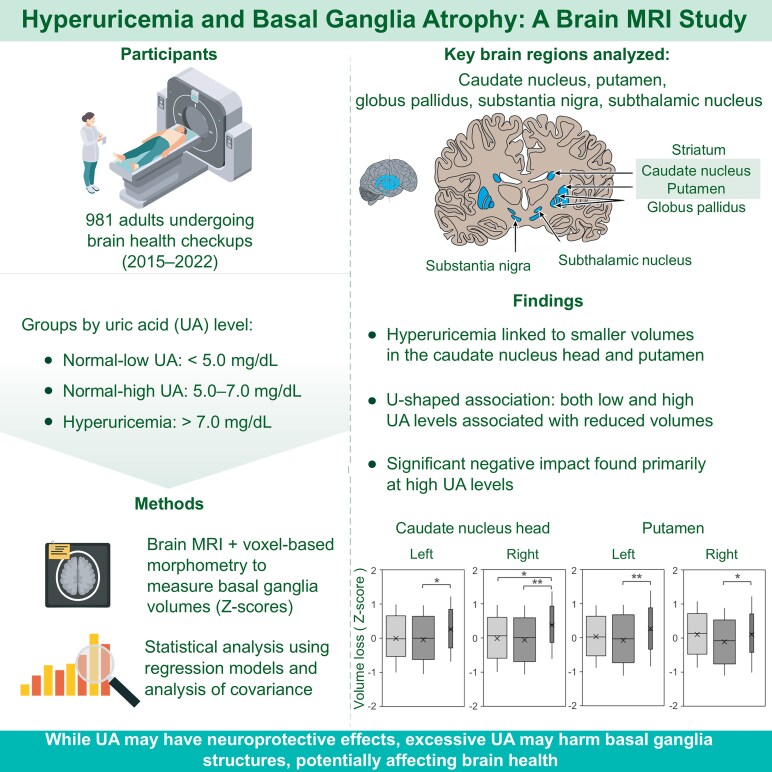

## Introduction

Uric acid (UA) is the end product of purine metabolism in humans, and hyperuricaemia occurs when the amount of UA that accumulates in the body exceeds the excretion thereof. Hyperuricaemia is a risk factor for gout, urinary tract stones, and kidney failure and threatens people’s health as a lifestyle-related disease.^1^ Studies have also identified the negative effects of hyperuricaemia on the central nervous system (CNS); for example, hyperuricaemia is independently associated with cerebrovascular disease, cognitive decline, and an enlarged perivascular space (EPVS) in the basal ganglia.^2-4^ From a biochemical perspective, UA crystals in the blood, which result from excess UA, have an inflammatory effect and may be involved in the promotion of neuronal damage and atherosclerosis.^5^

However, epidemiological data suggest that UA may have a protective effect on the CNS. Studies have reported lower UA levels in patients with Alzheimer’s disease and amyotrophic lateral sclerosis than in healthy controls.^6,7^ Furthermore, low UA levels contribute to the degeneration of dopaminergic neurons in Parkinson’s disease.^8^ These findings emphasize the neuroprotective effects of UA, based on experimental evidence that UA is a powerful antioxidant.^9^ Decreased UA levels result in elevated oxidative stress, which may be a risk factor for the progression of neurodegenerative diseases.^10^

Therefore, the potential effects of UA on the CNS remain controversial. In particular, it remains unclear whether UA is protective or toxic to the basal ganglia, a predilection site for cerebrovascular diseases and the responsible lesion in several neurodegenerative diseases such as Parkinson’s disease. We aimed to measure blood UA levels in community-dwelling adults who underwent brain health checkups and to evaluate their association with basal ganglia volume.

## Materials and methods

### Ethical statement and data source

**Figure 1:**
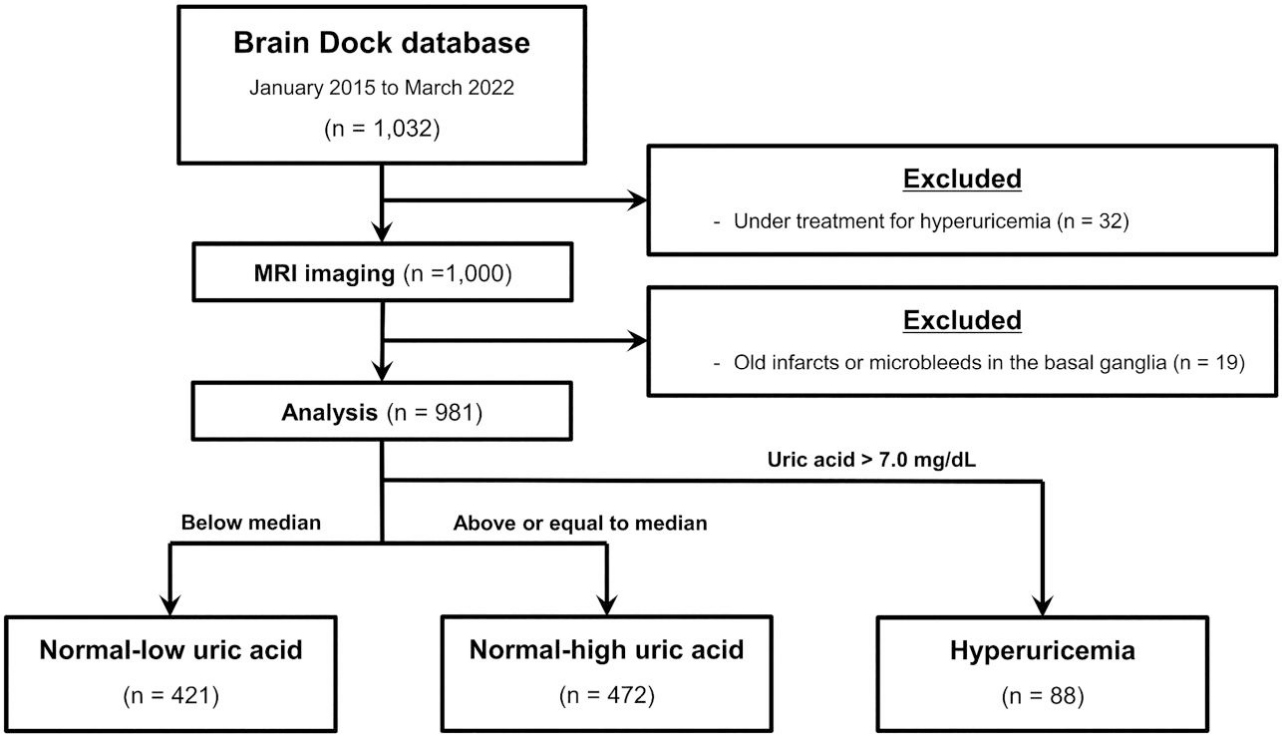
**Flow chart of study sample selection.** MRI, magnetic resonance imaging.

This retrospective study was approved by the Shimane University Ethics Committee (No. 20151028-1). Written informed consent was obtained from all the study participants in accordance with the Declaration of Helsinki. Brain health checkups using MRI and magnetic resonance angiography, commonly known as Brain Dock, have been widely performed in Japan since 1995.^11^ In addition to imaging studies, assessments included medical interviews, physical examinations, and laboratory-based testing. The participants were mainly middle-aged or older adults living in the local community, who underwent Brain Dock between January 2015 and March 2022 at the Shimane Institute of Health Science, Shimane, Japan. After excluding participants currently undergoing treatment for hyperuricaemia, 1000 participants were included for the evaluation of blood tests and neuroimaging data (Fig. 1).

### Clinical assessments

Trained nursing staff conducted structured interviews and standardized physical assessments. Participant characteristics, such as demographic details (age and sex), anthropometric data (height and weight), and relevant medical history (e.g. hypertension, diabetes, and dyslipidemia) were systematically recorded. Body mass index (BMI) was calculated by dividing body weight (in kilograms) by the square of height (in metres). Medical history was obtained through self-administered questionnaires and verified during clinical interviews. A history of hypertension, diabetes mellitus, or dyslipidemia was recorded as present if the participant was receiving treatment or had been formally diagnosed by a healthcare provider. Basic blood tests to assess the participants’ liver and kidney functions were routinely performed as part of a medical checkup in the Brain Dock. For participants who requested more detailed blood tests, plasma fibrinogen levels were measured as indicators of non-specific inflammatory response.

To minimize variability in the blood test results, the nurses obtained the participants’ blood samples in the morning after an 8–12 h overnight fast. Serum and plasma samples were centrifuged within 30 min in a central laboratory. Serum creatinine levels (µmol/L) were measured by the rate-blanked and compensated Jaffe creatinine method. Renal function was defined as the estimated glomerular filtration rate (eGFR), with levels (mL/min/1.73 m^2^) calculated based on the 2021 Chronic Kidney Disease Epidemiology Collaboration.^12^ Plasma fibrinogen levels (mg/dL) were measured using thrombin coagulation time method. Based on Japanese guidelines, hyperuricaemia was diagnosed when serum UA levels exceeded 7.0 mg/dL in both men and women.^13^

### Neuroimaging assessments

MRI was performed on the same day as the physical examination and blood tests using a Philips 3.0 T MRI system. The images were obtained using conventional T_1_-weighted, T_2_-weighted, fluid-attenuated inversion recovery (FLAIR), and $T_{2}^{*}$-weighted imaging. The acquisition parameters were as follows: T_1_-weighted, echo time (TR) = 7.35 ms, repetition time (TE) = 3.38 ms, field of view (FOV) 220 mm, matrix 244 × 164, slice thickness 2 mm; T_2_-weighted, TR = 2930.76 ms, TE = 80 ms, FOV 220 mm, matrix 512 × 300, slice thickness 6 mm; FLAIR, TR = 11 000 ms, TE = 120 ms, FOV 220 mm, matrix 340 × 246; $T_{2}^{*}$-weighted, TR = 540.56 ms, TE = 13.81 ms, FOV 220 mm, matrix 368 × 338, slice thickness 6 mm. Images were converted into the Neuroimaging Informatics Technology Initiative file format.

MRI scans were processed for Brain Anatomical Analysis using Diffeomorphic Deformation (BAAD, Shiga University of Medical Science, Shiga, Japan, version 4.4.0) software, designed to support VBM. VBM is a sophisticated technique that standardizes variables by normalising brain shape through coordinating transformations and adjusting for local volumes with covariate corrections at each voxel. This method facilitates the quantitative analysis of brain anatomy by allowing comparisons of the concentrations of brain tissue types (gray matter and white matter) across individuals. The procedural standards and methodological details of VBM have been extensively discussed in a previous publication.^14^ In summary, T_1_-weighted MRI images were set around the anterior and posterior commissure lines and resampled with a voxel size of 1 mm^3^. Subsequently, the images were segmented into gray matter, white matter, and cerebrospinal fluid, and warped into the Montreal Neurological Institute space, which was created as a new standard brain using a large series of MRI scans on healthy controls. Brain images reconstructed using Montreal Neurological Institute space were further segmented into anatomical volumes of interest (AVOI) in each hemisphere based on the automated anatomical labelling atlas.^15^ In this study, the basal ganglia were defined as the caudate nucleus head, caudate nucleus tail, lateral globus pallidus, medial globus pallidus, putamen, substantia nigra, and subthalamic nucleus. These structures were considered AVOIs, and volumes were calculated as standardized scores (*Z*-scores), adjusted for participant age, sex, and total intracranial volume. Further details on BAAD are available in the literature.^16^

In addition to basal ganglia volume measurements, the presence of asymptomatic infarcts and microbleeds was assessed according to the STRIVE-2 statement.^17^ Asymptomatic infarcts were defined as lesions >3 mm in size with a central hypointensity equivalent to that of cerebrospinal fluid and a hyperintense rim around the periphery. Microbleeds were characterized by blank signal areas with blooming on $T_{2}^{*}$-weighted images.

The perivascular spaces refer to ovoid or linear lesions that were visible as hypointense and hyperintense regions in the basal ganglia on T_1_ and T_2_-weighted images, respectively, and were considered ‘enlarged’ if their size was ≥ 2 mm. The guidelines of the Japanese Brain Dock Society established a classification based on the number of EPVS observed on a single slide at the basal ganglia level as follows: Grade 0, none; Grade 1, counts 1–5; Grade 2, counts 6–10; and Grade 3, counts ≥ 11.^18^ Skilled neuroradiologists performed the assessments.

### Covariates

Basal ganglia volumes were calculated as *Z*-scores after adjusting for participants’ age, sex, and total intracranial volume. BMI, eGFR, and the presence of lifestyle-related diseases such as hypertension, diabetes mellitus, and dyslipidemia, were also included in the analysis as covariates, in addition to age and sex. Based on the neuroimaging findings, the severity of EPVS in the basal ganglia was included as a covariate.

### Statistical analysis

As one of our goals was to evaluate differences between the hyperuricaemia and normal UA groups, participants were first classified according to their UA levels. To clarify the bidirectional effects of UA levels, the normal UA group (≤ 7.0 mg/dL) was divided into two additional groups, a normal-low UA group and a normal-high UA group, based on their median values. In addition to the hyperuricaemia group (> 7.0 mg/dL), three groups were included in the analysis. Descriptive statistics were used to summarize clinical characteristics, medical histories, laboratory data, and imaging data, including means, standard deviations and percentages. Categorical variables were compared using Fisher’s exact test, and continuous variables were compared using the Kruskal–Wallis test. Group differences in plasma fibrinogen levels and *Z*-scores of basal ganglia volumes were compared using analysis of covariance (ANCOVA). Tukey’s honest significant difference (HSD) test was used for multiple comparisons. A smooth-curve-fitting approach based on a generalized additive model was used to verify whether a linear or non-linear correlation exists between UA levels and basal ganglia volumes. If a non-linear association was confirmed, a piecewise linear regression model adjusted for covariates was used for each subgroup divided by the inflection point. To confirm the robustness of the results, a validation analysis was conducted by selecting only the participants who did not undergo detailed blood test and had missing plasma fibrinogen levels.

All analyses were conducted using statistical software packages R (http://www.R-project.org, The R Foundation) and Empower Stats (www.empowerstats.com; X&Y Solutions, Inc., Boston, MA, USA). Statistical significance was defined as a two-sided *P* value of < 0.05.

## Results

**Figure 2:**
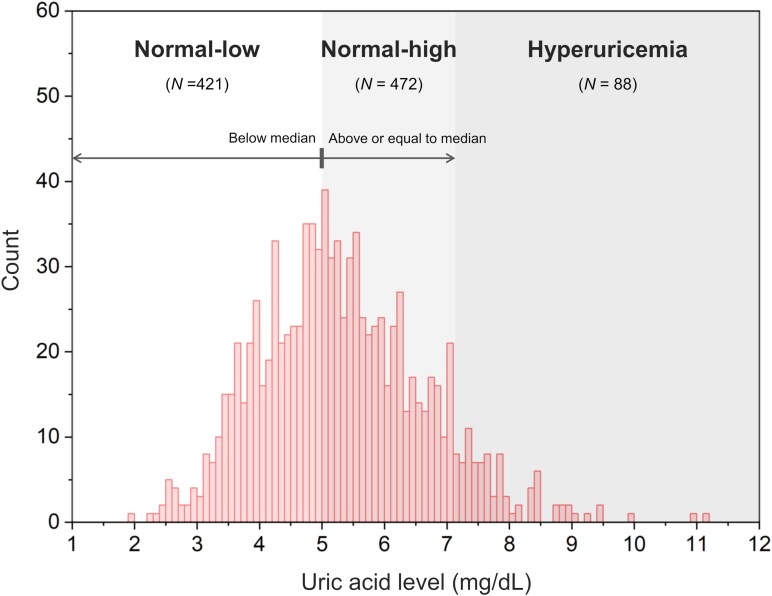
**Histogram showing distribution and classification of UA levels.** A Histogram illustrates the distribution of UA levels among 981 participants included in the analysis. The UA levels were divided into three categories: normal-low (1.9–4.9 mg/dL, *N* = 421) and normal-high (5.0–7.0 mg/dL, *N* = 472) groups based on the median UA value (5.0 mg/dL), and a hyperuricaemia group (> 7.0 mg/dL, *N* = 88). Bin width was set at 0.2 mg/dL.

Nineteen participants with infarcts or microbleeds in the basal ganglia on MRI were excluded, and 981 participants were included for analysis. Compared with the included participants, the excluded participants had a significantly higher mean age and greater prevalence of hypertension, whereas no significant difference in mean UA levels was observed (Supplementary Table 1). Of the participants included in the analysis, 88 met the criteria for hyperuricaemia. Among the 893 participants with normal UA levels, the lowest UA level was 1.9 mg/dL, and the highest was 7.0 mg/dL. The median UA level was 5.0 mg/dL. The participants with normal UA levels were further classified into two groups: normal-low UA group, 1.9–4.9 mg/dL; normal-high UA group, 5.0–7.0 mg/dL (Fig. 2). The baseline characteristics of the participants stratified by the UA group are shown in Table 1. The mean age of the participants was ∼60 years, with no statistically significant difference between the groups. The high UA group tended to have a larger proportion of men and a higher average BMI compared with the low UA group. Regarding medical history, significant group differences were observed in the history of hypertension and dyslipidemia. The mean eGFR filtration rate was significantly lower in the group with higher UA levels. Regarding the severity of EPVS, no overall significant group difference was found, although a trend toward a higher proportion of Grade 2 participants was observed in the group with higher UA levels.

**Table 1:** Characteristics of the participants stratified by serum UA levels

	Total (*N* = 981)	UA status			*P* value
		Normal-low UA (*N* = 421)	Normal-high UA (*N* = 472)	Hyperuricaemia (*N* = 88)	
UA, mean [range], mg/dL	5.27 [1.9–11.1]	4.10 [1.9–4.9]	5.83 [5.0–7.0]	7.86 [7.1–11.1]	
Clinical characteristics					
Age, mean (SD), year	61.1 (12.9)	61.1 (12.9)	61.4 (12.9)	60.0 (12.8)	0.595
Male sex, *N* (%)	516 (52.6)	114 (27.1)	325 (68.9)	77 (87.5)	**< 0.001**
BMI, mean (SD), kg/m^2^	22.7 (3.21)	21.7 (3.00)	23.2 (3.02)	24.6 (3.65)	**< 0.001**
Medical history, *N* (%)					
Hypertension	285 (29.1)	99 (23.5)	148 (31.4)	38 (43.2)	**< 0.001**
Diabetes mellitus	61 (6.2)	23 (5.5)	35 (6.0)	3 (3.4)	0.299
Dyslipidemia	209 (21.3)	96 (22.8)	104 (22.0)	9 (10.2)	**0.020**
Laboratory data, mean (SD)					
eGFR, mL/min/1.73 m^2^	71.2 (12.7)	73.5 (12.6)	70.2 (12.4)	64.9 (12.4)	**< 0.001**
Imaging data, *N* (%)					
EPVS					0.264
Grade 0	409 (41.7)	186 (44.2)	188 (39.8)	35 (39.8)	
Grade 1	470 (47.9)	199 (47.3)	232 (49.2)	39 (44.3)	
Grade 2	66 (6.7)	21 (5.0)	34 (7.2)	11 (12.5)	
Grade 3	36 (3.7)	15 (3.6)	18 (3.8)	3 (3.4)	

Characteristics of the participants stratified by serum UA levels. Clinical, laboratory, and neuroimaging characteristics of the 981 participants are presented according to UA classification: normal-low UA (1.9–4.9 mg/dL), normal-high UA (5.0–7.0 mg/dL), and hyperuricaemia (> 7.0 mg/dL). Differences among the groups were assessed using Fisher’s exact test for categorical variables and the Kruskal–Wallis test for continuous variables. Values in bold are statistically significant at *P* < 0.05. BMI, body mass index; eGFR, estimated glomerular filtration rate; EPVS, enlarged perivascular space; SD, standard deviation; UA, uric acid.

**Figure 3:**
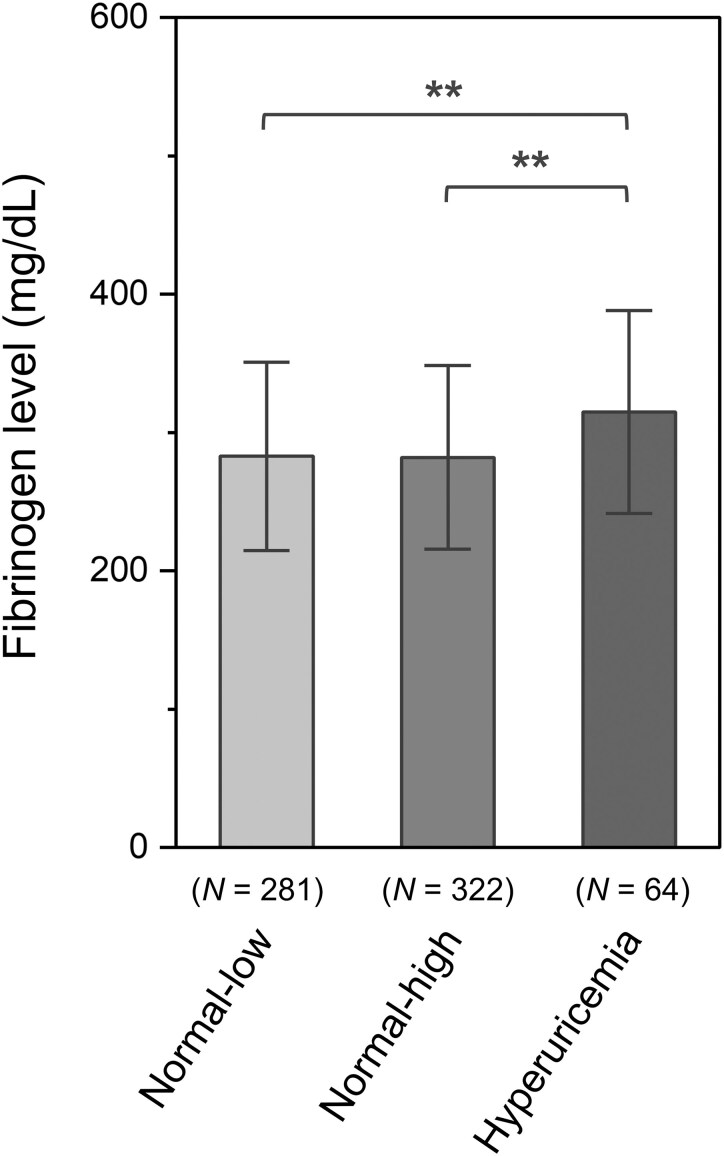
**Bar graph showing the mean plasma fibrinogen level for each UA level group.** Bar graphs and error bars indicate mean ± standard deviation. ANCOVA was performed to assess differences between the three UA level groups. The model was adjusted for participant age, sex, BMI, eGFR, history of hypertension, diabetes mellitus, dyslipidemia, and severity of EPVSs [*F*(2, 656) = 10.504, *P* < 0.001]. The *P* values were adjusted using Tukey’s HSD test. ** *P* < 0.01.

**Figure 4:**
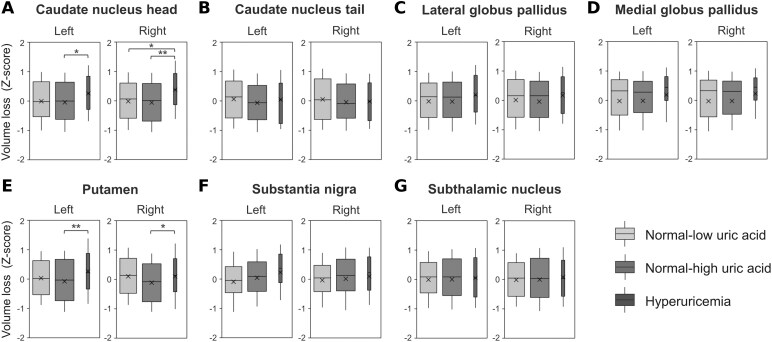
**Boxplots of basal ganglia volumes between UA level groups.** A total of 981 participants were included in the analysis. The boxplots represent the statistical distribution of the volumes of the caudate nucleus head (**A**), caudate nucleus tail (**B**), lateral globus pallidus (**C**), medial globus pallidus (**D**), putamen (**E**), substantia nigra (**F**), and subthalamic nucleus (**G**), categorized by UA groups. The volumes were adjusted for age, sex, and total intracranial volume and standardized as *Z*-scores, with a mean of zero and standard deviation of 1. Higher *Z*-scores indicate smaller brain volumes. Each box contains the middle 50% of the distribution; a horizontal line in the box represents the median, and a cross represents the mean. The *P* values calculated using the ANCOVA were adjusted using Tukey’s HSD test. * *P* < 0.05, ** *P* < 0.01.

The results of the ANCOVA and multiple comparisons, which evaluated the differences in fibrinogen levels among the groups, are presented in Supplementary Tables 2–4. After adjusting for covariates, the mean fibrinogen level was significantly higher in the hyperuricaemia group than in the other groups (Fig. 3). ANCOVA was also performed to assess group differences in the volumes of specific basal ganglia regions (Fig. 4A–G and Supplementary Table 5). Significant group differences were observed in the *Z*-scores of the bilateral caudate nucleus head and putamen volumes (Fig. 4A and E). Multiple comparison tests were performed only for the bilateral caudate nucleus head and putamen as post-hoc analyses. The hyperuricaemia group exhibited significant increases in the *Z*-score (i.e. smaller volume) compared with the normal-high UA group after Tukey’s HSD test (Supplementary Table 6). In the right caudate nucleus head, the hyperuricaemia group had significantly higher *Z*-scores than those of the normal-low and normal-high UA groups.

**Figure 5:**
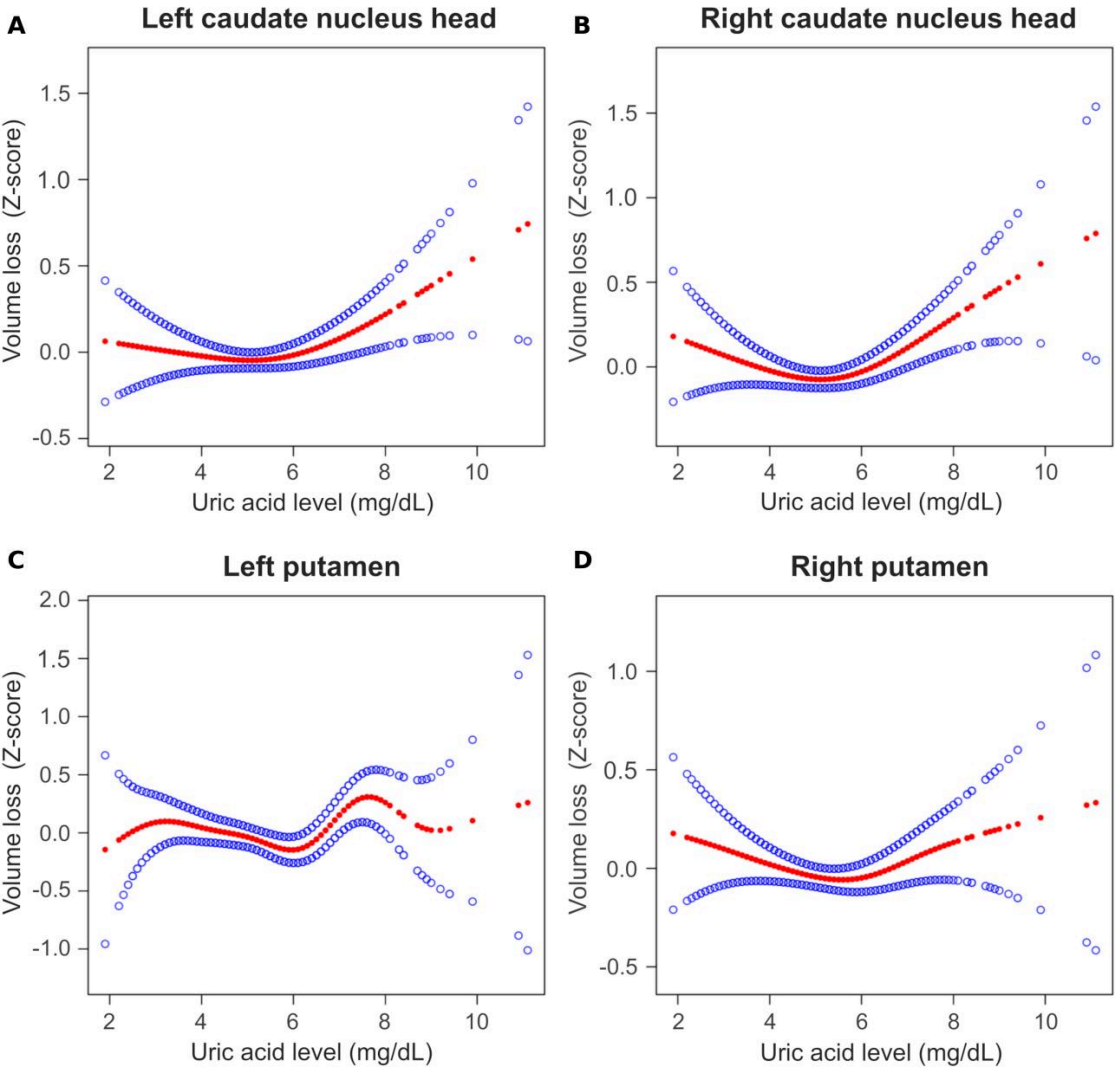
**Smooth-curve fit showing the relationship between UA levels and volumes of the bilateral caudate nucleus head and putamen.** A total of 981 participants were included in the analysis. The graphs show the continuous association between UA levels and the volumes of the left caudate nucleus head (**A**), right caudate nucleus head (**B**), left putamen (**C**), and right putamen (**D**). The volumes of the caudate nucleus head and putamen were adjusted for age, sex, and total intracranial volume and standardized as *Z*-scores, with a mean of zero and standard deviation of one. Higher *Z*-scores indicate smaller brain volume. Smooth curves were fitted based on a generalized additive model and adjusted for participant age, sex, BMI, eGFR, history of hypertension, diabetes mellitus, dyslipidemia, and severity of EPVSs. Points with filled markers represent the estimated volumes at each UA level, calculated from the fitted curve, and points with open markers indicate the 95% confidence intervals of these estimates.

These results indicate the possibility of negative correlations between UA levels and caudate nucleus head and putamen volumes. However, based on the results of multiple comparisons, a U-shaped association was also assumed, because the lowest mean *Z*-scores were observed in the normal-high UA group rather than in the normal-low UA group. Therefore, smooth-curve fitting was performed to visualize the relationship between UA levels as continuous variables and the volumes of the bilateral caudate nucleus head and putamen (Fig. 5A–D). In all the fitting curves, the inflection points of UA levels that resulted in the smallest volumes were identified in all the fitting curves (left caudate head: UA = 5.1 mg/dL, right caudate head: UA = 5.1 mg/dL, left putamen: UA = 6.0 mg/dL, and right putamen: UA = 5.6 mg/dL). The results of the piecewise linear regression conducted after categorisation into two groups around these inflection points are shown in Table 2. All models showed negative correlations with volumes in the lower UA group and positive correlations with volumes in the higher UA group, with significant regression lines observed only in the higher UA group.

**Table 2:** Multiple linear regression analysis for each category dichotomized by inflection points of UA levels

		Dependent variables			
		Caudate nucleus head, L	Caudate nucleus head, R	Putamen, L	Putamen, R
Categories					
UA ≤ inflection point	Β (95% CI)	−0.093 (−0.235–0.049)	−0.124 (−0.264–0.017)	−0.072 (−0.166–0.021)	−0.069 (−0.175–0.037)
	*P* value	0.198	0.085	0.129	0.199
UA ≥ inflection point	Β (95% CI)	0.164 (0.066–0.261)	0.198 (0.101–0.296)	0.216 (0.053–0.380)	0.179 (0.050–0.308)
	*P* value	**0.001**	**< 0.001**	**0.010**	**0.007**

Piecewise linear regression models were conducted for participants divided into lower and higher UA groups, using inflection points estimated from the smooth fitting curves for each brain region volume. The models assessed the association between UA levels and the *Z*-score standardized volumes of the bilateral caudate nucleus head and putamen. All models were adjusted for age, sex, BMI, eGFR, history of hypertension, diabetes mellitus, dyslipidemia, and severity of EPVSs. Values in bold are statistically significant at *P* < 0.05. CI, confidence interval; L, left; R, right; UA, uric acid.

We conducted a validation analysis to confirm the robustness of our results. Of all the participants, those whose plasma fibrinogen levels were measured were excluded, and ANCOVA was performed for participants who did not undergo detailed blood tests and had no data on plasma fibrinogen levels (*N* = 314). A total of 24 participants met the criteria for hyperuricaemia, while 290 participants with normal UA levels were classified into two groups based on the median value described above: normal-low UA group (1.9–4.9 mg/dL, *N* = 140); and normal-high UA group (5.0–7.0 mg/dL, *N* = 150). The results of the ANCOVA and multiple comparisons for this population are presented in Supplementary Tables 7 and 8. Similar to the analysis of the entire sample, significant group differences were found in the bilateral caudate nucleus heads and putamen. Multiple comparisons showed significantly higher Z-scores for the bilateral caudate nucleus heads in the hyperuricaemia group than in the other groups. In the right putamen, no significant difference in the volume was noted between the hyperuricaemia and normal-high UA groups, whereas the normal-low UA group had a significantly higher *Z*-score than that of the normal-high UA group.

## Discussion

We examined the relationship between blood UA levels and basal ganglia volume using a cross-sectional analysis. As the participants in this study were community-dwelling individuals who underwent brain health checkups, the distribution of UA levels was more reflective of the general population. ANCOVA revealed that plasma fibrinogen levels were significantly higher in the hyperuricaemia group than in the normal-low and normal-high UA level groups, and that the volumes of the bilateral caudate nucleus head and putamen were significantly smaller in the hyperuricaemia group than in the normal-high UA level group. Furthermore, a U-shaped association was suggested between the UA levels and the volumes of the bilateral caudate nucleus head and putamen. In the validation analysis, volume differences were observed in the same regions as in the analysis of the entire sample, confirming the robustness of the results. These results provide new evidence for conflicting hypotheses regarding the effects of UA on the basal ganglia.

Our epidemiological data on hyperuricaemia were generally consistent with previous findings. Participants diagnosed with hyperuricaemia were more likely to be male and had a significantly increased mean BMI. One reason for the sex difference in the prevalence of hyperuricaemia is that sex hormones, such as oestrogen and testosterone, control UA excretion.^19,20^ Therefore, the potential impact of hyperuricaemia on basal ganglia volumes identified in this study may be more critical for men, considering the higher prevalence of hyperuricaemia in males. However, susceptibility to hyperuricaemia is not determined by sex alone. A large Taiwanese population study reported that hyperuricaemia was associated with obesity-related indices and was more strongly associated with obesity in women than in men. In other words, obesity is an independent risk factor for hyperuricaemia, and hyperuricaemia can be considered a lifestyle-related disease, regardless of sex. Hyperuricaemia can lead to various complications, of which renal dysfunction is the most common.^21^ Gouty kidney, a condition marked by crystallized UA deposits in the kidney resulting in tissue damage and intravascular inflammation, serves as the primary cause of hyperuricaemia, leading to renal dysfunction.^22^ In our study, plasma fibrinogen levels, which reflect non-specific intravascular inflammation, were significantly higher only in the hyperuricaemia group. Considering that the limiting solubility of UA in plasma is ∼7 mg/dL, our findings may well reflect the biological aspects of UA.^23^

The effects of UA on the CNS have been investigated from various perspectives. Hyperuricaemia may be a risk factor for EPVS in the basal ganglia of patients with a history of lacunar infarction.^4^ Although no significant differences were detected in our data, EPVS tended to be more severe in the group with higher UA levels. As EPVS reflects inflammation of the small arteries and increased permeability of the blood–brain barrier, hyperuricaemia may exacerbate EPVS severity due to intravascular inflammation.^24^ However, high UA levels may be a factor that inhibits neuronal damage in certain neurodegenerative diseases.^25^ For example, low UA levels are associated with the early onset of motor and non-motor symptoms and striatal dopaminergic neuron activity in Parkinson’s disease.^8,26^ These associations have traditionally been attributed to the antioxidant effects of UA.^27^ Thus, our main question was whether a neuroprotective effect of UA could be observed in hyperuricaemia. The present analysis showed that the volumes of the bilateral caudate nucleus head and putamen were smaller in the hyperuricaemia group. All results were adjusted for the severity of EPVS, suggesting that hyperuricaemia may be associated with a smaller volume in the bilateral caudate nucleus head and putamen beyond the apparent volume reduction caused by EPVS.

Although the biological mechanism by which the high concentrations of UA impair the CNS remains unclear, several biochemical hypotheses have been proposed. For example, hyperuricaemia may act as a pro-oxidant, generating reactive oxygen species (ROS) and free radicals by inhibiting nitric oxide.^28^ In addition, elevated levels of xanthine oxidase, which are activated during UA metabolism, may produce ROS beyond the physiological range.^29^ These findings provide evidence supporting the possibility that UA levels above the solubility limit are harmful to the basal ganglia. Nevertheless, the smooth curves of the volumes in the caudate nucleus head and putamen showed a U-shaped association with a slight decrease in volume in the lower UA group. This U-shaped association suggests not only CNS damage associated with hyperuricaemia but also a possible contribution of insufficient neuroprotective effects when UA levels are low. However, in the piecewise linear regression analysis, a significant linear association between UA levels and the volumes of the caudate nucleus head and putamen was observed only in the higher UA group. This finding may be explained by the presence of a clear biochemical mechanism underlying hyperuricaemia-induced CNS damage, namely the accumulation of UA beyond its solubility limit in the blood.^30^ In contrast, although the insufficient neuroprotective effect at low UA levels has been supported by several epidemiological studies, the underlying biochemical pathways are more complex and heterogeneous.^31^ Such complexity may lead to greater individual variability and consequently reduce the statistical power to detect significant associations. A U-shaped association of UA levels has also been identified with other health outcomes, such as all-cause and cardiovascular mortality.^32^ Similarly, in the context of the CNS, maintaining UA levels within an intermediate range, rather than at excessively high or low levels, may provide the greatest benefit for neurological integrity.

A notable finding of this study was the association between hyperuricaemia and smaller volumes of the caudate nucleus head and putamen. These regions are anatomically referred to as the striatum and are innervated by dopaminergic neurons originating from the substantia nigra compacta and ventral tegmental area.^33^ Selective atrophy of the putamen has been observed in advanced Parkinson’s disease and the Parkinsonian variant of multiple system atrophy, possibly reflecting the degeneration of dopaminergic neurons.^34,35^ A smaller basal ganglia volume and reduced levels of dopamine transporters have also been reported in Lesch–Nyhan syndrome, a congenital form of hyperuricaemia.^36-38^ These studies have led to the hypothesis that dopaminergic neurons are particularly vulnerable to metabolic abnormalities. For example, dopaminergic neurons contain large amounts of iron that catalyze the production of oxidant molecules, and the enzymatic metabolism of dopamine via monoamine oxidase produces hydrogen peroxide as a byproduct.^39^ Considering the anatomical sites showing volume reductions, our analysis implies the possibility that hyperuricaemia-related metabolic disturbances in the CNS may promote vulnerability of dopaminergic neurons. However, since the present study did not focus on the degeneration of dopaminergic neurons, we cannot argue that the reduced striatal volume was due to a decrease in dopaminergic neurons. Further studies are required to determine whether hyperuricaemia contributes to the vulnerability of dopaminergic neurons in the general population. The caudate nucleus and putamen are easily damaged because of the higher incidence of EPVS and other small-vessel lesions.^40^ Although cases with evidence of old infarcts and microbleeds in the basal ganglia were excluded from the analysis, we cannot rule out the possibility that the high incidence of small-vessel lesions, especially in the putamen, may obscure the U-shaped association with UA levels.

The strength of this study is that UA levels were measured in community-dwelling participants with untreated hyperuricaemia, allowing for more clinically based assessments of the association between UA levels and basal ganglia volume. However, this study had some limitations. First, this was a cross-sectional study, and the association between UA levels and basal ganglia volume does not necessarily imply a causal relationship. Second, although this study was based on a community-dwelling sample, exclusion criteria were applied during the analytical process. Among the participants excluded from the study, those with infarcts or microbleeds in the basal ganglia may have exhibited more severe clinical features, whereas those receiving treatment for hyperuricaemia may have had milder imaging findings compared with the untreated group. These exclusion criteria may have introduced selection bias and limited the generalisability of the results. Third, lifestyle factors that may influence UA levels, such as alcohol consumption, were not sufficiently evaluated in this study. The presence of such unmeasured confounders should also be acknowledged as a limitation. Fourth, this study did not assess the severity of the participants’ clinical symptoms, particularly Parkinsonism. As a result, clinical manifestations associated with the decreased caudate nucleus head and putamen volumes were unclear. Recent studies using the VBM method have demonstrated a close relationship between Parkinson’s disease and basal ganglia atrophy.^41^ Clinical symptoms such as extrapyramidal signs may serve as valuable outcome measures comparable to structural brain atrophy. Finally, only a few participants presented with severe hypouricaemia in this study. The pathogenesis of hypouricaemia involves genetic abnormalities in the transporters responsible for UA reabsorption, and its prevalence is much lower than that of hyperuricaemia.^42^ Therefore, classifying hypouricaemia according to biochemical criteria is difficult, and low UA levels in this study were the only relative indicators in the general population.

In summary, we measured serum UA levels and basal ganglia volumes in participants undergoing brain health checkups and evaluated their associations. The caudate nucleus head and putamen volumes were significantly lower in the hyperuricaemia group than in the normal-high UA group. Although previous studies have supported the antioxidant effects of UA, untreated hyperuricaemia may have more disadvantages than benefits in the basal ganglia.

## Supplementary Material

fcaf263_Supplementary_Data

## Data Availability

The data are not available for ethical reasons. Further inquiries can be directed to the corresponding author. The code generated for this study can be found at https://github.com/NaokiOMORI-hub.
