# Supplementary material for: Hyperuricaemia is associated with smaller volumes in the caudate nucleus head and putamen
Source: Brain Commun. 2025 Jul 15;7(4):fcaf263. doi: 10.1093/braincomms/fcaf263 (PMC12260223; doi:10.1093/braincomms/fcaf263)
Supplement: fcaf263_Supplementary_Data [file fcaf263_supplementary_data.docx]

**Supplementary materials**

**Supplementary Table 1. Characteristics of the nineteen participants excluded from the analysis**

|  | **Excluded participants**  **(*N* = 19)** | **Total**  **(*N* = 981)** | ***p* value** |
| --- | --- | --- | --- |
| UA, mean (SD), mg/dL | 5.10 (1.1) | 5.27 (1.3) | 0.629 |
| Clinical characteristics |  |  |  |
| Age, mean (SD), year | 71.9 (10.2) | 61.1 (12.9) | **< 0.001** |
| Male sex, *N* (%) | 11 (57.9) | 516 (52.6) | 0.817 |
| BMI, mean (SD), kg/m^2^ | 23.6 (4.16) | 22.7 (3.21) | 0.321 |
| Medical history, *N* (%) |  |  |  |
| Hypertension | 11 (57.9) | 285 (29.1) | **0.010** |
| Diabetes mellitus | 3 (15.8) | 61 (6.2) | 0.116 |
| Dyslipidemia | 3 (15.8) | 209 (21.3) | 0.778 |
| Laboratory data, mean (SD) |  |  |  |
| eGFR, mL/min/1.73m^2^ | 67.7 (12.0) | 71.2 (12.7) | 0.132 |

Clinical and laboratory characteristics of the nineteen participants excluded due to infarcts or microbleeds in the basal ganglia are presented alongside those of the 981 included participants. The two groups were compared using Fisher’s exact test for categorical variables and the Mann–Whitney U test for continuous variables. BMI, body mass index; eGFR, estimated glomerular filtration rate; SD, standard deviation; UA, uric acid.

**Supplementary Table 2. Plasma fibrinogen level for each uric acid level group.**

|  | **Normal-low UA** | **Normal-high UA** | **Hyperuricemia** |
| --- | --- | --- | --- |
|  | **(*N* = 281)** | **(*N* = 332)** | **(*N* =64)** |
| Laboratory data, mean (SD) |  |  |  |
| Fibrinogen, mg/dL | 283 (68.2) | 282 (66.3) | 315 (73.3) |

Mean plasma fibrinogen levels are presented according to UA classification: normal-low UA (1.9–4.9 mg/dL), normal-high UA (5.0–7.0 mg/dL), and hyperuricemia (> 7.0 mg/dL). SD, Standard deviation; UA, uric acid.

**Supplementary Table 3. ANCOVA for plasma fibrinogen levels between uric acid level groups.**

|  | **Model SS** | **Model Df** | **Model MS** | **Residual SS** | **Residual Df** | **Residual MS** | **F value** | ***p* value** |
| --- | --- | --- | --- | --- | --- | --- | --- | --- |
| Fibrinogen | 84786.6 | 2 | 42438.3 | 2650000 | 656 | 4040.3 | 10.504 | **< 0.001** |

Mean plasma fibrinogen levels were compared across UA level groups (normal-low, normal-high, and hyperuricemia) using ANCOVA, adjusting for age, sex, body mass index, history of hypertension, diabetes mellitus, dyslipidemia, and severity of enlarged perivascular spaces. ANCOVA, analysis of covariance; Df, degrees of freedom; MS, mean square; SS, Sum of Square.

**Supplementary Table 4. Multiple comparisons for plasma fibrinogen levels between uric acid level groups**

| **Group (I)** | **vs** | **Group (J)** | **Mean difference (I-J)** | **Standard error of difference** | **Df** | **t value** | **Adjusted *p* value** |
| --- | --- | --- | --- | --- | --- | --- | --- |
|  |  |  |  |  |  |  |  |
| Normal-low UA |  | Normal-high UA | -3.91 | 6.08 | 656 | -0.643 | 0.796 |
|  |  | Hyperuricemia | -43.42 | 10.14 | 656 | -4.283 | **< 0.001** |
| Normal-high UA |  | Hyperuricemia | -39.51 | 8.95 | 656 | -4.415 | **< 0.001** |

The *p* values calculated using the analysis of covariance were adjusted using Tukey’s honest significant difference test. Df, degrees of freedom; UA, uric acid.

**Supplementary Table 5.** **ANCOVA for mean volumes in the basal ganglia between uric acid level groups.**

| Anatomical regions | **Model SS** | **Model Df** | **Model MS** | **Residual SS** | **Residual Df** | **Residual MS** | **F value** | ***p* value** |
| --- | --- | --- | --- | --- | --- | --- | --- | --- |
| Caudate nucleus head, L | 6.616 | 2 | 3.081 | 932.654 | 969 | 0.962 | 3.201 | **0.041** |
| Caudate nucleus head, R | 11.032 | 2 | 5.516 | 935.043 | 969 | 0.965 | 5.716 | **0.003** |
| Caudate nucleus tail, L | 3.738 | 2 | 1.869 | 954.000 | 969 | 0.985 | 1.898 | 0.150 |
| Caudate nucleus tail, R | 2.495 | 2 | 1.247 | 937.991 | 969 | 0.968 | 1.289 | 0.276 |
| Lateral globus pallidus, L | 3.541 | 2 | 1.770 | 945.094 | 969 | 0.975 | 1.815 | 0.163 |
| Lateral globus pallidus, R | 3.349 | 2 | 1.675 | 953.717 | 969 | 0.984 | 1.701 | 0.183 |
| Medical globus pallidus, L | 3.183 | 2 | 1.591 | 968.085 | 969 | 0.999 | 1.593 | 0.204 |
| Medical globus pallidus, R | 4.534 | 2 | 2.267 | 970.004 | 969 | 1.001 | 2.265 | 0.104 |
| Putamen, L | 9.707 | 2 | 4.854 | 923.005 | 969 | 0.952 | 5.096 | **0.006** |
| Putamen, R | 6.823 | 2 | 3.412 | 916.564 | 969 | 0.946 | 3.607 | **0.028** |
| Substantia nigra, L | 1.192 | 2 | 0.596 | 933.485 | 969 | 0.963 | 0.619 | 0.539 |
| Substantia nigra, R | 1.095 | 2 | 0.548 | 951.002 | 969 | 0.981 | 0.558 | 0.572 |
| Subthalamic nucleus, L | 0.187 | 2 | 0.094 | 925.811 | 969 | 0.955 | 0.098 | 0.906 |
| Subthalamic nucleus, R | 0.938 | 2 | 0.469 | 928.803 | 969 | 0.959 | 0.489 | 0.613 |

Adjusted mean Z-scores of basal ganglia volumes were compared across UA level groups (normal-low, normal-high, and hyperuricemia) using ANCOVA. Volumes were adjusted for age, sex, and total intracranial volume. The models also included covariates such as age, sex, body mass index, history of hypertension, diabetes mellitus, dyslipidemia, and severity of enlarged perivascular spaces. ANCOVA, analysis of covariance; Df, degrees of freedom; L, left; MS, mean square; R, right; SS, Sum of Square.

**Supplementary Table 6. Multiple comparisons for basal ganglia volumes between uric acid level groups**

|  | **Group (I)** | **vs** | **Group (J)** | **Mean difference (I-J)** | **Standard error of difference** | **Df** | **t value** | **Adjusted *p* value** |
| --- | --- | --- | --- | --- | --- | --- | --- | --- |
| Caudate nucleus head, L |  |  |  |  |  |  |  |  |
|  | Normal-low UA |  | Normal-high UA | 0.031 | 0.073 | 969 | 0.418 | 0.908 |
|  |  |  | Hyperuricemia | -0.265 | 0.127 | 969 | -2.085 | 0.093 |
|  | Normal-high UA |  | Hyperuricemia | -0.295 | 0.117 | 969 | -2.530 | **0.031** |
| Caudate nucleus head, R |  |  |  |  |  |  |  |  |
|  | Normal-low UA |  | Normal-high UA | 0.063 | 0.074 | 969 | 0.849 | 0.672 |
|  |  |  | Hyperuricemia | -0.332 | 0.129 | 969 | -2.586 | **0.027** |
|  | Normal-high UA |  | Hyperuricemia | -0.395 | 0.117 | 969 | -3.365 | **0.002** |
| Putamen, L |  |  |  |  |  |  |  |  |
|  | Normal-low UA |  | Normal-high UA | 0.105 | 0.073 | 969 | 1.43 | 0.328 |
|  |  |  | Hyperuricemia | -0.250 | 0.128 | 969 | -1.96 | 0.123 |
|  | Normal-high UA |  | Hyperuricemia | -0.355 | 0.117 | 969 | -3.04 | **0.007** |
| Putamen, R |  |  |  |  |  |  |  |  |
|  | Normal-low UA |  | Normal-high UA | 0.117 | 0.073 | 969 | 1.60 | 0.247 |
|  |  |  | Hyperuricemia | -0.160 | 0.127 | 969 | -1.26 | 0.420 |
|  | Normal-high UA |  | Hyperuricemia | -0.277 | 0.116 | 969 | -2.38 | **0.046** |

The *p* values calculated using the analysis of covariance were adjusted using Tukey’s honest significant difference test. Df, degrees of freedom; L, left; R, right; UA, uric acid.

**Supplementary Table 7. ANCOVA for the basal ganglia volumes between uric acid level groups in participants with no plasma fibrinogen level data.**

| Anatomical regions | **Model SS** | **Model Df** | **Model MS** | **Residual SS** | **Residual Df** | **Residual MS** | **F value** | ***p* value** |
| --- | --- | --- | --- | --- | --- | --- | --- | --- |
| Caudate nucleus head, L | 7.245 | 2 | 3.622 | 280.701 | 302 | 0.929 | 3.897 | **0.021** |
| Caudate nucleus head, R | 9.070 | 2 | 4.535 | 253.217 | 302 | 0.839 | 5.408 | **0.005** |
| Caudate nucleus tail, L | 4.886 | 2 | 2.443 | 307.891 | 302 | 1.020 | 2.396 | 0.093 |
| Caudate nucleus tail, R | 1.153 | 2 | 0.577 | 277.210 | 302 | 0.918 | 0.628 | 0.534 |
| Lateral globus pallidus, L | 1.383 | 2 | 0.692 | 265.385 | 302 | 0.879 | 0.787 | 0.456 |
| Lateral globus pallidus, R | 0.222 | 2 | 0.111 | 268.966 | 302 | 0.891 | 1.125 | 0.883 |
| Medical globus pallidus, L | 1.186 | 2 | 0.593 | 257.341 | 302 | 0.852 | 0.696 | 0.499 |
| Medical globus pallidus, R | 2.151 | 2 | 1.075 | 270.637 | 302 | 0.896 | 1.200 | 0.303 |
| Putamen, L | 6.625 | 2 | 3.312 | 271.229 | 302 | 0.898 | 3.688 | **0.026** |
| Putamen, R | 7.968 | 2 | 3.984 | 240.698 | 302 | 0.797 | 4.999 | **0.007** |
| Substantia nigra, L | 0.297 | 2 | 0.149 | 138.152 | 302 | 0.457 | 0.325 | 0.723 |
| Substantia nigra, R | 0.464 | 2 | 0.232 | 132.953 | 302 | 0.440 | 0.527 | 0.591 |
| Subthalamic nucleus, L | 0.733 | 2 | 0.367 | 176.404 | 302 | 0.584 | 0.628 | 0.535 |
| Subthalamic nucleus, R | 0.885 | 2 | 0.443 | 219.352 | 302 | 0.726 | 0.609 | 0.544 |

Adjusted mean Z-scores of basal ganglia volumes were compared across UA level groups (normal-low, normal-high, and hyperuricemia) using ANCOVA in 314 participants with missing plasma fibrinogen values. The models also included covariates such as age, sex, body mass index, history of hypertension, diabetes mellitus, dyslipidemia, and severity of enlarged perivascular spaces. ANCOVA, analysis of covariance; Df, degrees of freedom; L, left; MS, mean square; R, right; SS, Sum of Square.

**Supplementary Table 8. Multiple comparisons for the basal ganglia volumes between the four uric acid level groups in participants with no plasma fibrinogen level data.**

|  | **Group (I)** | **vs** | **Group (J)** | **Mean difference (I-J)** | **Standard error of difference** | **Df** | **t value** | **Adjusted *p* value** |
| --- | --- | --- | --- | --- | --- | --- | --- | --- |
| Caudate nucleus head, L |  |  |  |  |  |  |  |  |
|  | Normal-low UA |  | Normal-high UA | 0.024 | 0.120 | 302 | 0.197 | 0.979 |
|  |  |  | Hyperuricemia | -0.587 | 0.230 | 302 | -2.558 | **0.030** |
|  | Normal-high UA |  | Hyperuricemia | -0.611 | 0.221 | 302 | -2.770 | **0.016** |
| Caudate nucleus head, R |  |  |  |  |  |  |  |  |
|  | Normal-low UA |  | Normal-high UA | 0.055 | 0.114 | 302 | 0.483 | 0.879 |
|  |  |  | Hyperuricemia | -0.633 | 0.218 | 302 | -2.903 | **0.011** |
|  | Normal-high UA |  | Hyperuricemia | -0.688 | 0.209 | 302 | -3.329 | **0.003** |
| Putamen, L |  |  |  |  |  |  |  |  |
|  | Normal-low UA |  | Normal-high UA | 0.166 | 0.188 | 302 | 1.408 | 0.338 |
|  |  |  | Hyperuricemia | -0.387 | 0.226 | 302 | -1.716 | 0.201 |
|  | Normal-high UA |  | Hyperuricemia | -0.553 | 0.217 | 302 | -2.553 | **0.030** |
| Putamen, R |  |  |  |  |  |  |  |  |
|  | Normal-low UA |  | Normal-high UA | 0.325 | 0.111 | 302 | 2.922 | **0.010** |
|  |  |  | Hyperuricemia | -0.034 | 0.213 | 302 | -0.160 | 0.986 |
|  | Normal-high UA |  | Hyperuricemia | -0.359 | 0.204 | 302 | -1.756 | 0.186 |

The *p* values calculated using the analysis of covariance were adjusted using Tukey’s honest significant difference test. Df, degrees of freedom; L, left; R, right; UA, uric acid.
